# Association of dietary ω-3 and ω-6 fatty acids intake with cognitive performance in older adults: National Health and nutrition examination Survey (NHANES) 2011–2014

**DOI:** 10.1186/s12937-020-00547-7

**Published:** 2020-03-28

**Authors:** Xue Dong, Shiru Li, Jiahao Chen, Yan Li, Yanjun Wu, Dongfeng Zhang

**Affiliations:** Department of Epidemiology and Health Statistics, School of Public Health, Qingdao University, 38 Deng Zhou Street, Qingdao, 266021 Shandong Province China

**Keywords:** Cognitive performance, Dietary ω-3 fatty acids, Dietary ω-6 fatty acids, ω-6: ω-3 ratio, Dose-response

## Abstract

**Background:**

Current evidence on the association of dietary ω-3 and ω-6 fatty acids intake with cognitive performance is inconsistent. Therefore, the aim is to explore the association of dietary ω-3 and ω-6 fatty acids intake with cognitive performance in the U.S. noninstitutionalized population of older adults.

**Methods:**

We used data from the National Health and Nutrition Examination Survey (NHANES) 2011–2014. Intakes of ω-3 and ω-6 fatty acids were obtained through two 24-h dietary recalls and were adjusted by energy. Cognitive performance was evaluated by the Consortium to Establish a Registry for Alzheimer’s disease (CERAD) Word Learning sub-test, Animal Fluency test and Digit Symbol Substitution Test (DSST). For each cognitive test, people who scored lower than the lowest quartile in each age group were defined as having low cognitive performance. Binary logistic regression and restricted cubic spline models were applied to evaluate the association of dietary ω-3 and ω-6 fatty acids intake with cognitive performance.

**Results:**

A total of 2496 participants aged 60 years or older were included. In the full-adjusted model, the odds ratios (ORs) with 95% confidence interval (CI) of CERAD test score, Animal Fluency test score and DSST test score were 0.58 (0.38–0.88), 0.68 (0.47–0.99) and 0.59 (0.37–0.92) for the highest versus lowest tertile of dietary ω-3 fatty acids intake, respectively; the ORs with 95% CI of CERAD test score, Animal Fluency test score and DSST test score were 0.48 (0.31–0.75), 0.60 (0.40–0.92) and 0.50 (0.34–0.75) for the highest versus lowest tertile of dietary ω-6 fatty acids intake, respectively. The association between ω-6: ω-3 ratio and cognitive performance was not statistically significant in three tests. In dose-response relationship analysis, L-shaped associations were apparent for ω-3 and ω-6 fatty acids intake with CERAD test score, Animal Fluency test score and DSST test score.

**Conclusions:**

Dietary ω-3 and ω-6 fatty acids intake might be inversely associated with low cognitive performance.

## Introduction

As life expectancy increases, the number of elderly people is growing worldwide. It is estimated that the number of the elderly aged 65 years or older in the U.S. grows by 15%, and the number of the elderly aged 85 years or older grows even more by 30% between 2000 and 2010 [1]. Age-related cognitive decline can be a major health challenge for the elderly population, cognitive health has emerged as an important public health concern for America’s aging population [2]. In America, approximately 36% of people are cognitively impaired, and 5.1 million people have dementia, with an expected doubling by 2050 [3]. The financial burden of dementia has already far exceeded the costs of cardiovascular and cancer diseases [4, 5]. In 2015, the global cost of dementia was $9575.6 billion and it is estimated to reach $2.54 trillion in 2030 and $9.12 trillion in 2050 [6]. The irreversibility of dementia, the lack of effective treatment and heavy financial burden make it imperative to prevent and treat low cognitive performance. Thus, developing measures to reduce the prevalence of low cognitive performance as well as treatments of diagnosed dementia have high priority in society [7].

Previous studies have shown that cognitive performance was determined by both genetic and environmental factors [8–15]. For instance, fruits and vegetables [16], vitamin D [13], vitamin B, protein, folate [11, 12] and polyphenols [17] have been proved to have protective effects on cognitive function, whereas some studies [14, 15] indicated that high level of saturated fat and refined sugar were associated with impaired cognitive performance.

Polyunsaturated fatty acids(PUFAs)are essential to human body functions because they can improve the activity of brain cells, enhance the memory and thinking abilities, and help reduce cardiovascular and cerebrovascular diseases [18–20]. But some studies have pointed out that ω-3 and ω-6 fatty acids intake may play a role in the pathogenesis of cognitive decline due to the prevailing mechanisms such as oxidative stress, inflammation, and vascular risk factors [21, 22]. However, several studies have suggested that ω-3 and ω-6 fatty acids and their ratio could be associated with cognitive performance among elderly people [23–25]. Specifically, a low ratio of ω-6 to ω-3 fatty acids in the diet might reduce the risk of cognitive decline [26, 27].

To date, epidemiological studies which investigated the association of ω-3 and ω-6 fatty acids intake with cognitive performance in older Americans are scarce during NHANES examination in 2011–2012 and 2013–2014, and the results for the association of ω-3 and ω-6 fatty acids intake with cognitive performance are not entirely consistent [28–31]. Therefore, we analyzed a dataset of Americans aged 60 years or older from NHANES to investigate the association of ω-3 and ω-6 fatty acids intake and ω-6: ω-3 ratio with cognitive performance.

## Materials and methods

### Data collection and study population

The NHANES is a 2-year-cycle cross-sectional survey conducted by the Centers for Disease Control and Prevention (CDC) of America, which aims to evaluate the health and nutritional status of the U.S. population. Representative samples of the non-institutional U.S. population are selected by a complex multi-stage probabilistic sampling design. Participants were interviewed at home firstly and then completed the health examination in a mobile examination center (MEC) [32]. The NHANES database is a publicly available dataset used by people worldwide [33]. All participants have provided informed consent both before the interview and examination stages.

**Fig. 1:**
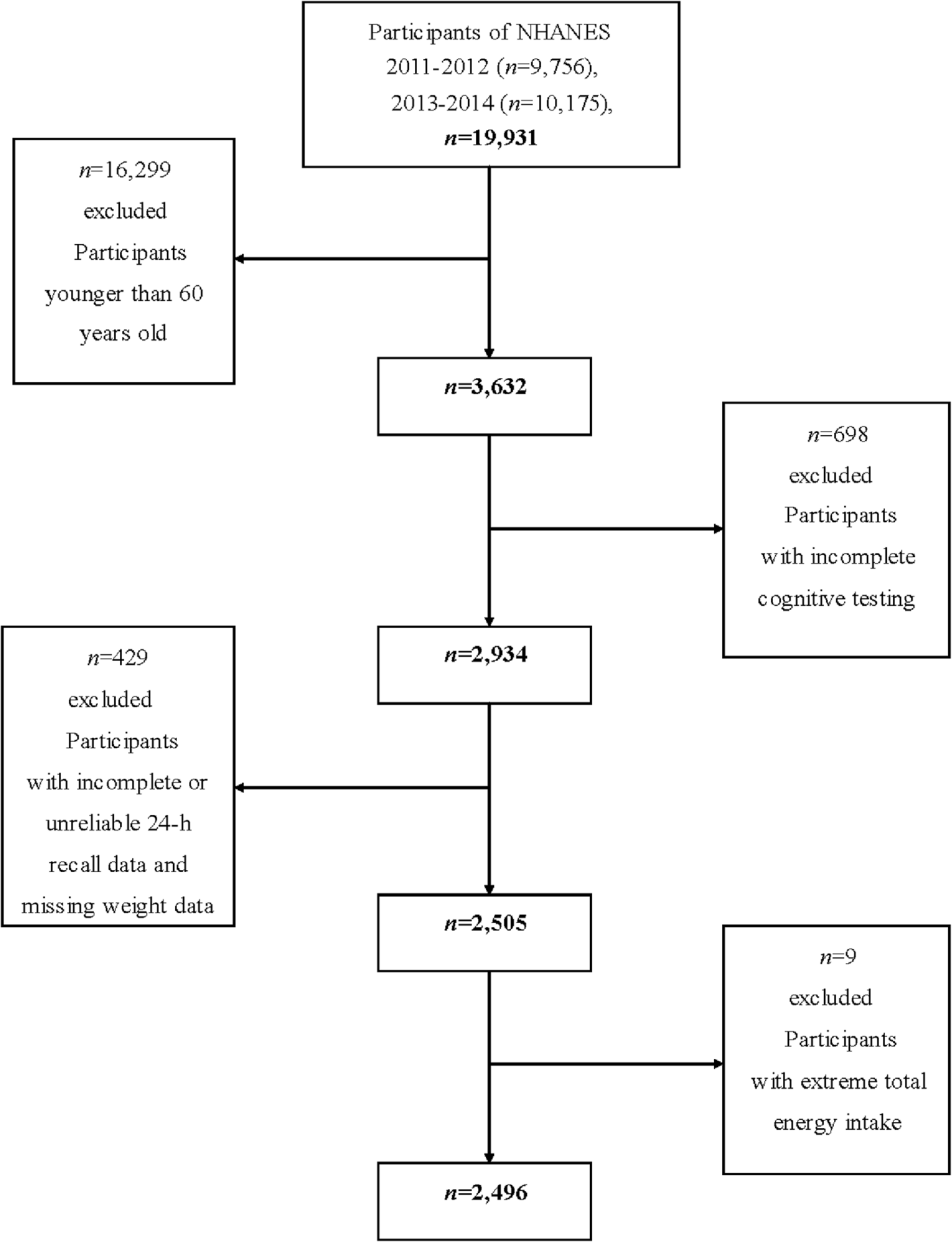
Flow chart of the screening process for the selection of eligible participants

A total of 19,931 individuals participated in the NHANES during 2011–2014, and our analyses were limited to 3632 individuals aged 60 years or older. Among them, we excluded participants with incomplete cognitive testing (*n* = 698), incomplete or unreliable 24-h recall data (*n* = 410) and missing weight data (*n* = 19). Individuals who had extreme total energy intakes of < 500 or > 5000 kcal/day for women, and < 500 or > 8000 kcal/day for men (*n* = 9) were further omitted. After exclusions, this study contained a total of 2496 participants aged 60 years or older (1204 men and 1292 women) (Fig. 1).

### Cognitive performance assessment

A series of assessments for cognitive performance in NHANES were used in 2011–2014 and cognitive testing was performed among participants aged 60 years or older [32]. Cognitive performance was assessed in a household interview or a Mobile Examination Center (MEC) and was evaluated by the Consortium to Establish a Registry for Alzheimer’s disease (CERAD) Word Learning sub-test, the Animal Fluency test and the Digit Symbol Substitution Test (DSST). These tests, which have been used in large screenings, epidemiological and clinical studies [34–39], evaluated working memory, language, processing speed, and executive functioning in older adults. Participants were asked for consent to audio-record the administration for quality control purpose. For interviews in English and Spanish, two interviewers transcribed verbatim responses from the audio recordings and scored the CERAD test, Animal fluency test and DSST test. Transcription and scoring usually were done on the same day assessments were conducted. Tests conducted in Korean, Vietnamese and Chinese were transcribed verbatim and scored by consultants in those languages later. Inconsistent scores were adjudicated by a third party, as necessary. In addition, review of the audio-recordings of assessments were evaluated for consistency of interviewer instructions and to determine test score accuracy. Approximately 10% of recorded interviews were independently reviewed during data collection [40].

The CERAD test consisted of three consecutive learning trials as well as a delayed recall, which were designed to assess immediate and delayed learning ability for new verbal information, respectively. For the learning trials, participants were organized to read aloud 10 unrelated words when they were presented one at a time. Immediately following the introduction of the words, participants recalled as many words as possible. The order of the 10 words was altered in each of the three learning trials. The delayed word recall occurred after Animal Fluency and DSST tests were completed. The score on each trial ranged from 0 to 10, and the total score of the CERAD test was the sum of three learning trials and a delayed recall trial. As a component of executive function, the Animal Fluency test examined categorical verbal fluency, participants were called upon to name as many animals as possible in 1 min. The score was the sum for the number of correct answers. The DSST test, a performance module from the Wechsler Adult Intelligence Scale, was used to assess processing speed, sustained attention and working memory. The exercise was performed using a piece of paper with a key at the top pairing numbers with nine symbols. Participants have 2 min to copy the corresponding symbols from the key into 133 numbered boxes. The score, ranging from 0 to 133, was the sum for the number of correct matches. Higher scores were linked to better cognitive performance for all tests.

Currently, there is not a gold standard cut-off point for the CERAD, DSST and Animal Fluency test to identify low cognitive performance. Therefore, we used the 25th percentile of the score, the lowest quartile, as the cut-off point, which is consistent with methods used in the published literature [41]. Furthermore, considering that age has a significant effect on cognitive performance, the score was further categorized based on age (60 to < 70 years, 70 to < 80 years, and ≥ 80 years). For each cognitive test, people who scored lower than the lowest quartile in each age group were defined as having low cognitive performance.

### Dietary intake assessment

Dietary ω-3 and ω-6 fatty acids intakes were obtained from two 24-h dietary recall interviews. The primary dietary interview was collected in-person in the mobile examination center (MEC), a follow-up dietary interview was conducted by telephone from the home office. Trained interviewers conducted dietary recall interviews using an automated data collection system during the MEC examination. At the end of the MEC dietary interview, the interviewers would schedule the participants for a phone follow-up (PFU) interview 3–10 days later. Dietary telephone interviewers at the home office would conduct the PFU interviews. Detailed descriptions of dietary interview and data processing procedures could be found under the dietary interview components on the NHANES website [42].

Ultimately, in our analyses, octadecatrienoic acid (18:3), octadecatetraenoic acid (18:4), eicosapentaenoic acid (20:5), docosapentanoic acid (22:5), and docosahexaenoic acid (22:6) were included in ω-3 fatty acids, meanwhile octadecadienoic acid (18:2) and eicosatetraenoic acid (20:4) were included in ω-6 fatty acids [43]. The average daily ω-3 and ω-6 fatty acids intake were calculated according to the U.S. Department of Agriculture’s Dietary Study Food and Nutrition Database for Dietary Studies [44] and were adjusted to total energy. Dietary ω-3 and ω-6 fatty acids intake (mg/kcal/day) and ω-6: ω-3 ratio were divided into tertiles, ω-3 and ω-6 intake was calculated from dietary intake only, supplement use was not collected in the NHANES 2011–2014.

### Covariates

In addition to dietary ω-3 and ω-6 fatty acids intake, we investigated some potential confounding factors, which included: age (60–70 years, 70–80 years, and ≥ 80 years), gender (Male and Female), race (Mexican American, other Hispanic, Non-Hispanic White, Non-Hispanic Black, and other race), educational level (Below high school, High school, and Above high school), marital status (Married/Living with partner and Widowed/Divorced/Separated/Never married), body mass index (BMI) (normal: < 25 kg/m^2^; overweight: 25 to < 30 kg/m^2^; obese: ≥30 kg/m^2^) and drinking (Having at least 12 alcohol drinks per year or not).

The physical activity questionnaire is based on the Global Physical Activity Questionnaire (GPAQ), and the questions were asked using the computer-assisted personal interview software [45]. The main outcomes of physical activity were defined using the following questions: (1) Vigorous work activity: “Does your work involve vigorous-intensity activity that causes large increases in breathing or heart rate like carrying or lifting heavy loads, digging or construction work for at least 10 minutes continuously?” (yes, no); (2) Moderate work activity: “Does your work involve moderate-intensity activity that causes small increases in breathing or heart rate such as brisk walking or carrying light loads for at least 10 minutes continuously?” (yes, no); (3) Vigorous recreational activity: “Do you do any vigorous-intensity sports, fitness, or recreational activity that cause large increases in breathing or heart rate like running or basketball for at least 10 minutes continuously?” (yes, no); (4) Moderate recreational activity: “Do you do any moderate-intensity sports, fitness, or recreational activity that cause a small increase in breathing or heart rate such as brisk walking, bicycling, swimming, or volleyball for at least 10 minutes continuously?” (yes, no). Participants were labelled as “vigorous work activity” if the answer for question (1) was yes, “moderate work activity” if the answer for question (1) was no and question (2) was yes, “other” if the answer for question (1) and question (2) were both no. The same was true for recreational activity. Poverty income ratio (PIR) — the ratio of family income to the poverty threshold — was used to define income. The Department of Health and Human Services (HHS) poverty guidelines were used as the poverty measure to calculate this ratio [46]. PIR was divided into two levels (≤0.99, ≥1). A ratio of 0.99 or less indicated that family income was below the poverty level, while a ratio of 1 or greater implied that family income was at or above the poverty level. History of hypertension, diabetes or stroke was defined as self-reported physician diagnosis of hypertension, diabetes or stroke.

### Statistical analysis

All statistical analyses were conducted with Stata 15.0 (Stata Corporation, College Station, TX). Account for the complex sampling design and ensure nationally representative estimates, all analyses were adjusted for survey design and weighting variables. Because we combine two cycles of the NHANES data, new sample weights (the original 2-year sample weight divided by 2) was constructed according to the analytical guidelines of the NHANES [47]. Kolmogorov-Smirnov normality test was adopted to test the normality of continuous variables and we described normally distributed variables with mean ± standard deviation, non-normal distributed variables with median (interquartile range). Student’s t-tests was utilized to compare the mean levels between low cognitive performers and not low cognitive performers if the variable was normally distributed, otherwise, the Mann-Whitney U test was adopted. When comparing the percentages of categorical variables between low cognitive performers and not low cognitive performers, we choose Chi-square tests. Dietary ω-3 and ω-6 fatty acids intake and ω-6: ω-3 ratio were categorized based on tertiles (tertile 1: <33th percentile, tertile 2: ≥33 to 67th percentile, tertile 3: ≥67th percentile) with tertile 1 as the reference category. Cognitive performance was analyzed as a binary variable. We conducted binary logistic regression analyses to examine the association of dietary ω-3 and ω-6 fatty acids intake and ω-6: ω-3 ratio with cognitive performance. Model 1 adjusted for age and gender. Model 2 was additionally adjusted for race, educational level, marital status, poverty income ratio, body mass index, recreational activity, work activity, drinking status, diabetes, hypertension and stroke. We analyzed the multicollinearity of the logistic regression. The tolerance was greater than 0.1, and the variance inflation factor (VIF) was less than 10, so there was no multicollinearity. We further used restricted cubic spline with 3 knots located at the 5th, 50th, and 95th percentiles of the exposure distribution to assess dose-response relationship in the logistic regression model 2. The *p*-value for non-linearity was calculated by testing the value of the coefficient of the second spline of zero [48]. A two-sided *p* < 0.05 was considered statistically significant.

## Results

Table 1 presents the characteristics of the study population across cognitive status. Ultimately, a total of 2496 participants were included in the study. There were significant differences between people with low cognitive performance and normal cognitive performance in the distribution of race, educational level, poverty income ratio, work activity, recreational activity, stroke, diabetes and total ω-6 fatty acids intake among CERAD test, Animal Fluency test and DSST test. People with low cognitive performance tended to have lower educational level, poverty income ratio, work activity, recreational activity, total ω-6 fatty acids intake, and higher prevalence of stroke and diabetes than people with normal cognitive performance. Higher age was associated with lower cognitive performance in Animal Fluency test. The prevalence of hypertension in people with low cognitive performance with Animal Fluency and DSST test was significantly higher than that of people with normal cognitive performance. Alcohol drinking rate was significantly lower in people with low cognitive performance with Animal Fluency and DSST test than people with normal cognitive performance. People with low cognitive performance with CERAD and DSST test were more likely to be male and have less ω-3 fatty acids intake. People with low cognitive performance with CERAD test tended to have lower ratio of ω-6 to ω-3 fatty acids.

**Table 1:** Characteristics of the study population, NHANES 2011–2014 (*N* = 2496)

	CERAD test			Animal Fluency test			Digit Symbol test		
	Normal Cognitive Performance	Low Cognitive Performance	*P* Value	Normal Cognitive Performance	Low Cognitive Performance	*P* Value	Normal Cognitive Performance	Low Cognitive Performance	*P* Value
**Number of subjects (%)**^**1**^	1851(74.2)	645(25.8)		1778(71.2)	718(28.8)		1850(74.1)	646(25.9)	
**Age (%)**^**1**^			0.578			0.017			0.930
**60–70 years**	1017(54.9)	339(52.6)		956(53.8)	400(55.7)		1006(54.4)	350(54.2)	
**70–80 years**	540(29.2)	198(30.7)		552(31.0)	186(25.9)		549(29.7)	189(29.3)	
**≥ 80 years**	294(15.9)	108(16.7)		270(15.2)	132(18.4)		295(15.9)	107(16.6)	
**Gender (%)**^**1**^			< 0.01			0.767			< 0.01
**Male**	811(43.8)	393(60.9)		861(48.4)	343(47.8)		838(45.3)	366(56.7)	
**Female**	1040(56.2)	252(39.1)		917(51.6)	375(52.2)		1012(54.7)	280(43.3)	
**Race (%)**^**1**^			< 0.01			< 0.01			< 0.01
**Mexican American**	136 (7.3)	75(11.6)		149(8.4)	62(8.6)		120(6.5)	91(14.1)	
**Other Hispanic**	153 (8.3)	90(14.0)		150(8.4)	93(13.0)		119(6.4)	124(19.2)	
**Non-Hispanic White**	1002(54.1)	253(39.2)		1026(57.7)	229(31.9)		1087(58.8)	168(26.0)	
**Non-Hispanic Black**	399(21.6)	183(28.4)		328(18.4)	254(35.4)		342(18.5)	240(37.2)	
**Other race**	161(8.7)	44(6.8)		125(7.0)	80(11.1)		182(9.8)	23(3.6)	
**Educational level (%)**^**1**^			< 0.01			< 0.01			< 0.01
**Below high school**	344(18.6)	249(38.7)		327(18.4)	266(37.2)		243(13.1)	350(54.3)	
**High school**	432(23.4)	158(24.5)		401(22.6)	189(26.4)		439(23.7)	151(23.4)	
**Above high school**	1074(58.1)	237(36.8)		1050(59.1)	261(36.5)		1168(63.1)	143(22.2)	
**Material status (%)**^**1**^			0.675			0.050			< 0.01
**Married/Living with partner**	1089(58.9)	374(58.0)		1064(59.9)	399(55.6)		1140(61.7)	323(50.1)	
**Widowed/Divorced/Separated/Never married**	759(41.1)	271(42.0)		712(40.1)	318(44.4)		708(38.3)	322(49.9)	
**Poverty income ratio (%)**^**1**^			< 0.01			< 0.01			< 0.01
**≤ 0.99**	236(13.8)	128(21.6)		214(12.9)	150(23.0)		184(10.7)	180(30.5)	
**≥ 1**	1480(86.2)	464(78.4)		1442(87.1)	502(77.0)		1534(89.3)	410(69.5)	
**Body mass index (%)**^**1**^			0.092			0.638			0.498
**< 25 kg/m**^**2**^	476(25.9)	184(28.6)		464(26.1)	196 (27.7)		487(26.4)	173(27.2)	
**25–30 kg/m**^**2**^	629(34.2)	233(36.2)		625(35.2)	237(33.5)		653(35.4)	209(32.8)	
**≥ 30 kg/m**^**2**^	735(39.9)	226(35.1)		686(38.6)	275(38.8)		706(38.2)	255(40.0)	
**Work activity (%)**^**1**^			< 0.01			< 0.01			< 0.01
**Vigorous**	220 (11.9)	55(8.5)		216(12.1)	59(8.2)		223(12.1)	52(8.0)	
**Moderate**	403(21.8)	108(16.7)		389(21.9)	122(17.0)		411(22.2)	100(15.5)	
**Other**	1228(66.3)	482(74.7)		1173(66.0)	537(74.8)		1216(65.7)	494(76.5)	
**Recreational activities (%)**^**1**^			< 0.01			< 0.01			< 0.01
**Vigorous**	205(11.1)	36(5.6)		199(11.2)	42(5.8)		227(12.3)	14(2.2)	
**Moderate**	615(33.2)	202(31.3)		621(34.9)	196(27.3)		639(34.5)	178(27.6)	
**Other**	1031(55.7)	407(63.1)		958(53.9)	480(66.9)		984(53.2)	454(70.3)	
**Hypertension (%)**^**1**^	1149(62.1)	406(63.1)	0.641	1069(60.0)	486(67.8)	< 0.01	1114(60.3)	441(68.3)	< 0.01
**Diabetes (%)**^**1**^	412(22.3)	173(26.8)	0.019	374(21.0)	211(29.4)	< 0.01	372(20.1)	213(33.0)	< 0.01
**Had at least 12 alcohol drinks/1 yr (%)**^**1**^	1272(69.0)	446(70.2)	0.566	1262(71.4)	456(64.2)	< 0.01	1316(71.5)	402(63.1)	< 0.01
**Ever told you had a stroke (%)**^**1**^	108(5.9)	54(8.4)	0.025	99(5.6)	63(8.8)	0.003	89(4.8)	73(11.3)	< 0.01
**Total ω-3 fatty acids intake (mg/kcal/day)**^**2**^	0.87(0.50)	0.84(0.50)	0.014	0.88(0.50)	0.86(0.48)	0.156	0.88(0.50)	0.82(0.42)	< 0.01
**Total ω-6 fatty acids intake (mg/kcal/day)**^**2**^	7.83(3.65)	7.23(3.36)	< 0.01	7.80(3.72)	7.37(3.27)	0.002	7.87(3.76)	7.08(3.06)	< 0.01
**ω-6: ω-3 ratio**^**2**^	8.61(3.00)	8.32(2.57)	0.032	8.55(2.84)	8.44(3.04)	0.261	8.54(2.92)	8.45(2.74)	0.207

Data are number of subjects (percentage) or medians (inter quartile ranges)

^1^ Chi-square test was used to compare the percentage between participants with and without low cognitive performance

^2^ Mann-Whitney *U* test was used to compare the mean values between participants with and without low cognitive performance

Table 2 shows the associations between dietary ω-3 fatty acids intake, dietary ω-6 fatty acids intake, ω-6: ω-3 ratio and CERAD test score. The crude ORs with 95% CI of CERAD test score were 0.54 (0.38–0.77) and 0.41 (0.27–0.63) in the highest versus lowest tertile of dietary ω-3 and ω-6 fatty acids intake, respectively. After adjustment for age and gender, ω-3 and ω-6 fatty acids intake were still inversely associated with low cognitive performance. In model 2, the multivariate adjusted ORs with 95% CI of CERAD test score were 0.58 (0.38–0.88) and 0.48 (0.31–0.75) in the highest versus lowest tertile of dietary ω-3 and ω-6 fatty acids intake, respectively. The association between ω-6: ω-3 ratio and CERAD test score was not statistically significant.

**Table 2:** Weighted odds ratios (95% confidence intervals) for score on CERAD test across tertiles of dietary ω-3 and ω-6 fatty acids intake and ω-6: ω-3 ratio, NHANES 2011–2014 (*N* = 2496)

	Case/Participants	Crude^1^	Model 1^1^	Model 2^1^
ω-3 (mg/kcal/day)				
<0.727	229/832	1.00 (Ref.)	1.00 (Ref.)	1.00 (Ref.)
0.727 to <1.04	233/832	0.66(0.46–0.94) *	0.66(0.46–0.94) *	0.63(0.43–0.91) *
≥ 1.04	183/832	0.54(0.38–0.77) **	0.55(0.38–0.78) **	0.58(0.38–0.88) *
ω-6 (mg/kcal/day)				
<6.538	253/832	1.00 (Ref.)	1.00 (Ref.)	1.00 (Ref.)
6.538 to <8.848	217/832	0.55(0.39–0.79) **	0.56(0.39–0.80) **	0.57(0.38–0.86) **
≥ 8.848	175/832	0.41(0.27–0.63) **	0.44(0.28–0.67) **	0.48(0.31–0.75) **
ω-6: ω-3 ratio				
<7.684	228/832	1.00 (Ref.)	1.00 (Ref.)	1.00 (Ref.)
7.684 to <9.462	222/832	1.03(0.78–1.35)	1.05(0.80–1.37)	0.93(0.67–1.29)
≥ 9.462	195/832	0.89(0.60–1.31)	0.94(0.64–1.38)	1.02(0.65–1.58)

^1^Calculated using binary logistic regression

Model 1 adjusted for age and gender

Model 2 adjusted for age and gender, race, educational level, marital status, income, BMI, recreational activity, work activity, drinking status, hypertension, diabetes and stroke

*p < 0.05; **p < 0.01

The associations between dietary ω-3 fatty acids intake, dietary ω-6 fatty acids intake, ω-6: ω-3 ratio and Animal Fluency test score are presented in Table 3. In logistic regression analyses, the crude ORs with 95% CI of Animal Fluency test score were 0.68 (0.48–0.96) and 0.60 (0.43–0.84) in the highest versus lowest tertile of dietary ω-3 and ω-6 fatty acids intake, respectively. After adjustment for age and gender, ω-3 and ω-6 fatty acids intake were still inversely associated with low cognitive performance. In model 2, the multivariate adjusted ORs with 95% CI of Animal Fluency test score were 0.68 (0.47–0.99) and 0.60 (0.40–0.92) in the highest versus lowest tertile of dietary ω-3 and ω-6 fatty acids intake, respectively. The association between ω-6: ω-3 ratio and Animal Fluency test score was not statistically significant.

**Table 3:** Weighted odds ratios (95% confidence intervals) for score on Animal Fluency test across tertiles of dietary ω-3 and ω-6 fatty acids intake and ω-6: ω-3 ratio, NHANES 2011–2014 (*N* = 2496)

	Case/Participants	Crude^1^	Model 1^1^	Model 2^1^
ω-3 (mg/kcal/day)				
<0.727	245/832	1.00 (Ref.)	1.00 (Ref.)	1.00 (Ref.)
0.727 to <1.04	255/832	0.77(0.55–1.05)	0.75(0.54–1.03)	0.75(0.54–1.03)
≥ 1.04	218/832	0.68(0.48–0.96) *	0.67(0.48–0.94) *	0.68(0.47–0.99) *
ω-6 (mg/kcal/day)				
<6.538	262/832	1.00 (Ref.)	1.00 (Ref.)	1.00 (Ref.)
6.538 to <8.848	249/832	0.69(0.47–1.03)	0.69(0.46–1.03)	0.66(0.43–1.02)
≥ 8.848	207/832	0.60(0.43–0.84) **	0.61(0.43–0.87) **	0.60(0.40–0.92) *
ω-6: ω-3 ratio				
<7.684	245/832	1.00 (Ref.)	1.00 (Ref.)	1.00 (Ref.)
7.684 to <9.462	232/832	1.08(0.80–1.47)	1.09(0.81–1.47)	1.17(0.83–1.65)
≥ 9.462	241/832	1.01(0.70–1.45)	1.04(0.71–1.53)	1.15(0.73–1.81)

^1^Calculated using binary logistic regression

Model 1 adjusted for age and gender

Model 2 adjusted for age and gender, race, educational level, marital status, income, BMI, recreational activity, work activity, drinking status, hypertension, diabetes and stroke

*p < 0.05; **p < 0.01

Table 4 shows the associations between dietary ω-3 fatty acids intake, dietary ω-6 fatty acids intake, ω-6: ω-3 ratio and DSST test score. The crude ORs with 95% CI of DSST test score were 0.63 (0.43–0.91), 0.47 (0.32–0.68) and 0.71 (0.53–0.93) in the highest versus lowest tertile of ω-3, ω-6 fatty acids intake and ω-6: ω-3 ratio, respectively. After adjustment for age and gender, the results were similar to the crude ORs (95% CIs). In model 2, the multivariate adjusted ORs (95% CI) of DSST test score were 0.59 (0.37–0.92) and 0.50 (0.34–0.75) in the highest versus lowest tertile of dietary ω-3 and ω-6 fatty acids intake, respectively. The association between ω-6: ω-3 ratio and the DSST test score was not significant.

**Table 4:** Weighted odds ratios (95% confidence intervals) for score on Digit Symbol Substitution test across tertiles of dietary ω-3 and ω-6 fatty acids intake and ω-6: ω-3 ratio, NHANES 2011–2014 (*N* = 2496)

	Case/Participants	Crude^1^	Model 1^1^	Model 2^1^
ω-3 (mg/kcal/day)				
<0.727	243/832	1.00 (Ref.)	1.00 (Ref.)	1.00 (Ref.)
0.727 to <1.04	232/832	0.88(0.61–1.24)	0.86(0.60–1.21)	0.93(0.57–1.51)
≥ 1.04	171/832	0.63(0.43–0.91) *	0.62(0.42–0.88) *	0.59(0.37–0.92) *
ω-6 (mg/kcal/day)				
<6.538	254/832	1.00 (Ref.)	1.00 (Ref.)	1.00 (Ref.)
6.538 to <8.848	235/832	0.76(0.56–1.01)	0.76(0.57–1.02)	0.78(0.51–1.18)
≥ 8.848	157/832	0.47(0.32–0.68) **	0.49(0.33–0.71) **	0.50(0.34–0.75) **
ω-6: ω-3 ratio				
<7.684	223/832	1.00 (Ref.)	1.00 (Ref.)	1.00 (Ref.)
7.684 to <9.462	228/832	1.05(0.73–1.51)	1.06(0.74–1.52)	1.01(0.66–1.57)
≥ 9.462	195/832	0.71(0.53–0.93) *	0.74(0.55–0.99) *	0.86(0.54–1.36)

1Calculated using binary logistic regression

Model 1 adjusted for age and gender

Model 2 adjusted for age and gender, race, educational level, marital status, income, BMI, recreational activity, work activity, drinking status, hypertension, diabetes and stroke

*p < 0.05; **p < 0.01

**Fig. 2:**
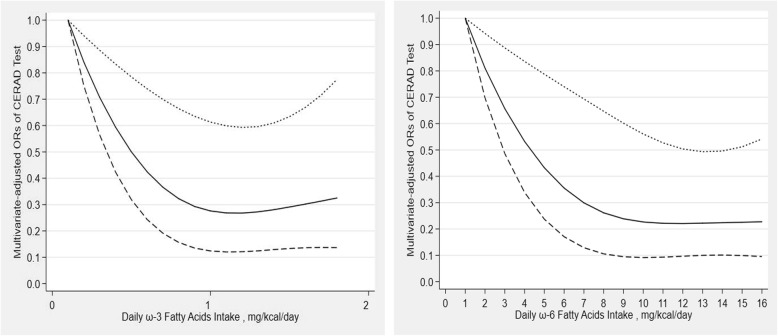
Dose-response relationship between ω-3 and ω-6 fatty acids intake and cognitive functioning in CERAD test. The association was adjusted for age, gender, race, educational level, marital status, income, BMI, recreational activity, work activity, drinking status, hypertension, diabetes and stroke. The solid line and dash line represent the estimated ORs and its 95% confidence intervals. (OR, odds ratio)

The results of the restricted cubic spline dose-response relationship analysis between dietary ω-3 fatty acids intake, dietary ω-6 fatty acids intake and CERAD test score were presented in Fig. 2. There was a nonlinear negative correlation and an L-shaped association between dietary ω-3 fatty acids intake and CERAD test score (P for nonlinearity = 0.041). Similarly, an L-shaped association between dietary ω-6 fatty acids intake and CERAD test score was also found.

**Fig. 3:**
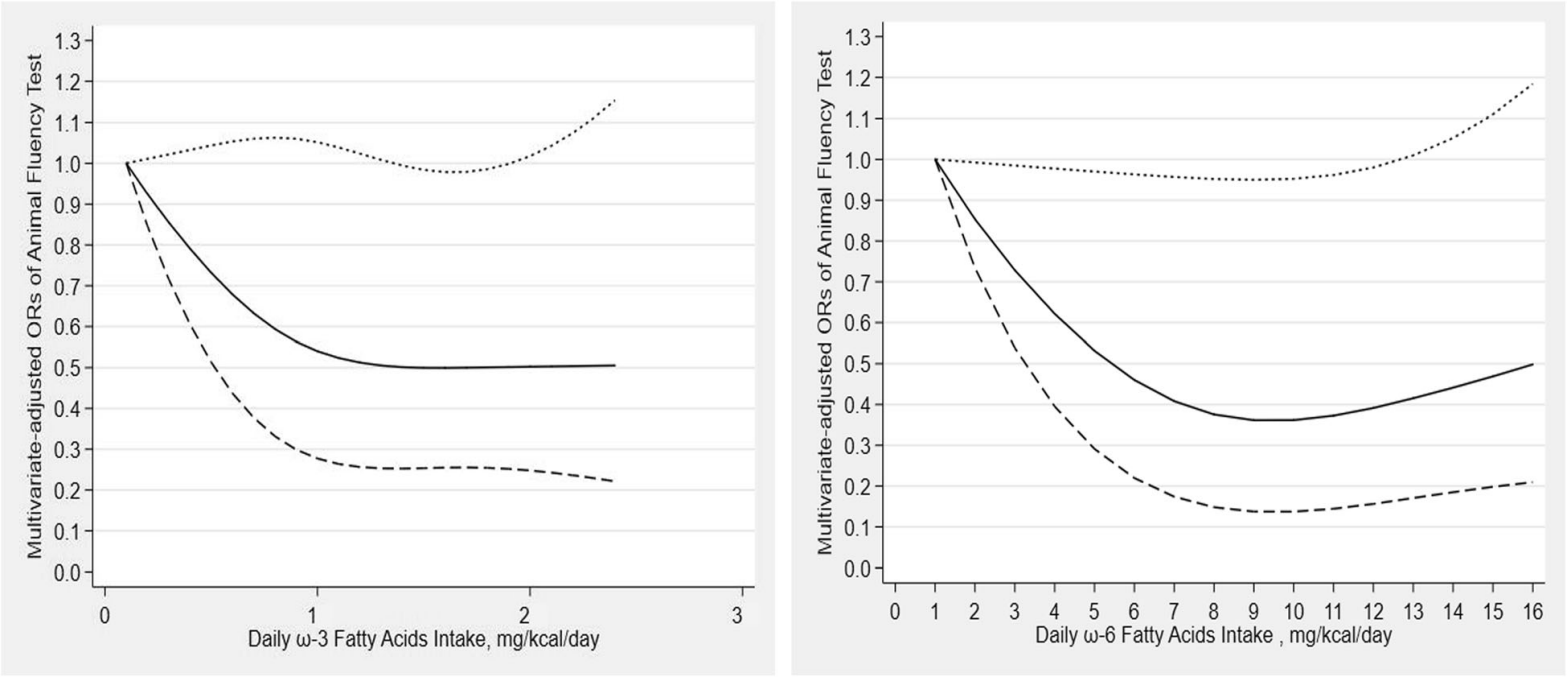
Dose-response relationship between ω-3 and ω-6 fatty acids intake and cognitive functioning in Animal Fluency test. The association was adjusted for age, gender, race, educational level, marital status, income, BMI, recreational activity, work activity, drinking status, hypertension, diabetes and stroke. The solid line and dash line represent the estimated ORs and its 95% confidence intervals. (OR, odds ratio)

The results of the restricted cubic spline dose-response relationship analysis between dietary ω-3 fatty acids intake, dietary ω-6 fatty acids intake and Animal Fluency test score were depicted in Fig. 3. There was an L-shaped correlation between dietary ω-3 fatty acids intake and Animal Fluency test score, and no significant association was observed in Animal Fluency test score with an intake lower than 1.5 mg/kcal/day (OR: 0.50; 95% CI: 0.25–0.99) or beyond 1.9 mg/kcal/day (OR: 0.50; 95% CI: 0.25–1.00). Meanwhile, there was also an L-shaped correlation between dietary ω-6 fatty acids intake and Animal Fluency test score, and there was significant association in Animal Fluency test score at < 12 mg/kcal/day (OR: 0.39; 95% CI: 0.16–0.99).

**Fig. 4:**
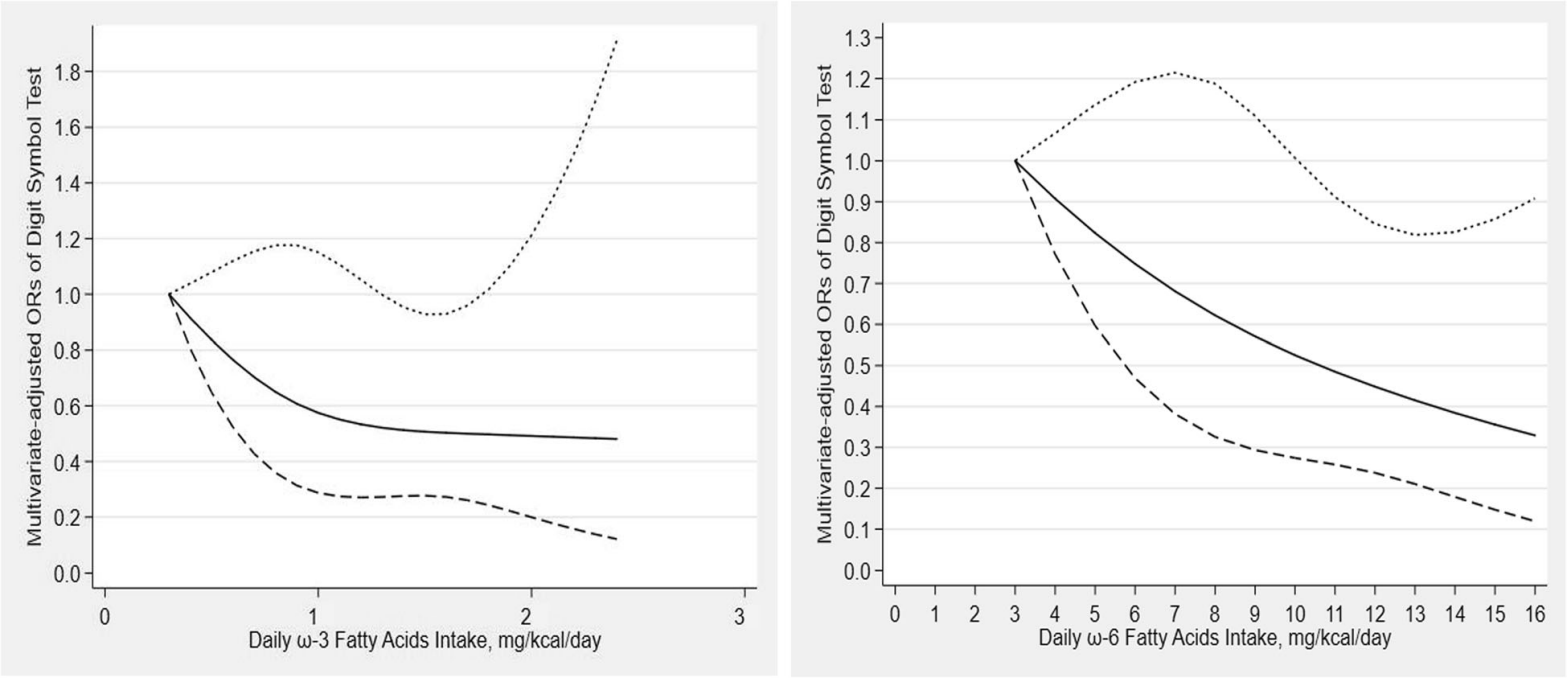
Dose-response relationship between ω-3 and ω-6 fatty acids intake and cognitive functioning in Digit Symbol test. The association was adjusted for age, gender, race, educational level, marital status, income, BMI, recreational activity, work activity, drinking status, hypertension, diabetes and stroke. The solid line and dash line represent the estimated ORs and its 95% confidence intervals. (OR, odds ratio)

The results of the restricted cubic spline analyses between dietary ω-3 fatty acids intake, dietary ω-6 fatty acids intake and DSST test score were depicted in Fig. 4. An L-shaped association between dietary ω-3 fatty acids intake and DSST test score was found. No significant association was observed in DSST test score with an intake lower than 1.3 mg/kcal/day (OR: 0.52; 95% CI: 0.27–1.00) or beyond 1.7 mg/kcal/day (OR: 0.50; 95% CI: 0.26–0.97). There was a linear negative correlation between dietary ω-6 fatty acids intake and DSST test score, and there was significant association in DSST test score at > 11 mg/kcal/day (OR: 0.48; 95% CI: 0.26–0.91).

## Discussion

Cognitive performance has important implications for the U.S. aging population. This study explores the associations of dietary ω-3 and ω-6 fatty acids intake and ω-6: ω-3 ratio with cognitive performance among the U.S. population aged 60 years or older. In the full-adjusted model, the highest versus lowest tertile of dietary ω-3 and ω-6 fatty acids intake were inversely associated with CERAD test score, Animal Fluency test score and DSST test score, respectively. The association between ω-6: ω-3 ratio and cognitive performance was not statistically significant in three tests. In dose-response relationship analysis, L-shaped associations were apparent for ω-3 and ω-6 fatty acids intake with CERAD test score, Animal Fluency test score and DSST test score.

Some studies use age- and education-specific cut points when identifying low cognitive performers [49]. Because cognitive performance on average declines with age, a lower score for an older person could be considered impairment, when in fact, it is within the normal range of performance for people of that age. However, this impairment would be missed if the cut-point score for the lowest 25th percentile is calculated without considering age. Therefore, in this report, low performers are defined by cut points that are conditional on age. Similarly, a lower score for a person with less education may be considered cognitive impairment, when in fact, it does not represent a decline from previous levels. Considering the issue with education, we put education in the covariates when performed a regression analysis. According to the objective of a study, researchers may choose to control various factors and use different cut points.

The results of our study were consistent with some previous studies [30, 50–53]. Fotuhi et al. [50] found the intake of ω-3 fatty acids could reduce the risk of cognitive decline in elderly individuals. Moreover, a 2-year follow-up study performed by Sandra Kalmijn et al. [51] as well as a 3-year follow-up study conducted by Beydoun [30] also indicated this negative association between ω-3 fatty acids and cognitive decline. Nevertheless, some results were inconsistent with previous studies. Some studies [29, 31] indicated that there was no significant association between ω-3 fatty acids intake and cognitive impairment. Our study found the intake of ω-6 fatty acids might be inversely associated with low cognitive performance, and the study conducted by Das et al. [53] also illuminated this negative association. In contrast, Arendash et al. [31] stated that high level of ω-6 fatty acids were associated with cognitive impairment. There was no significant association between the ratio of ω-6 to ω-3 fatty acids and cognitive performance in our study, whereas Andruchow et al. and Sheppard et al. [28, 54] found a low level of ω-6: ω-3 ratio in the diet might be inversely associated with the risk of cognitive decline in older adults.

The mechanisms of the relationship between dietary ω-3 and ω-6 fatty acids intake and cognitive performance remain unclear, but there have been several possibilities. First of all, ω-3 fatty acids may play an important role in the transmission of ions and neurotransmitters inside and outside of nerve cells [55]. If ω-3 fatty acids are insufficient, they will be replaced by other kinds of fatty acids, which may reduce the fluidity of the cell membrane, damage synapses and dendrites [56] and disrupt the concentration of neurotransmitters inside and outside of nerve cells, thus having a serious impact on brain function. Second, ω-3 fatty acids increase cell viability through neuro-protective and anti-apoptotic mechanisms [57], and they may reduce neuronal oxidative stress. Third, ω-3 fatty acids can promote neurogenesis and long term potential (LTP) formation in the hippocampus [58], and improve the function of the hippocampus. Calderon et al. [59] also found that ω-3 fatty acids could promote the survival of hippocampal neurons, and increase the number and length of neuronal processes significantly. Fourth, the ω-3 fatty acids may produce an anti-inflammatory effect [22], that is, they can reduce the production of pro-inflammatory cytokines in humans [60], and inflammatory reaction is of great significance in the development and progression of cognitive decline. The mechanism of ω-6 fatty acids is unclear. Therefore, further research is required to explore the relationship between ω-6 fatty acids intake and cognitive performance.

Our study presents several advantages. First, we used a large nationally representative sample of older adults in the US, which increased the statistical power to provide a more reliable result. Second, we investigated the dose-response relationship between dietary ω-3 and ω-6 fatty acids intake and cognitive performance. Third, we adjusted for some potential confounding factors in the process of exploring the associations of ω-3 and ω-6 fatty acids intake with cognitive performance.

Our study has several limitations. Primarily, as a cross-sectional study, it was difficult to ascertain causality. Furthermore, although we have adjusted for some confounders, we cannot rule out the potential confounding bias from unmeasured confounders. Participants who had mental or physical conditions that could influence performance, or used medications that may be associated with cognitive performance were not excluded from this analysis. What’s more, because NHANES data measured cognitive performance only at one point in time and in selected domains, these measures could not replace a diagnosis based on a clinical examination. The cognitive tests, chosen for ease of administration, availability, and use in other surveys, did not cover all domains of cognition. Adults who performed well in one domain may not perform well in another domain. Finally, the dietary data were obtained from two 24-h dietary recall interviews, which could not accurately reflect individuals’ usual intake.

## Conclusions

In conclusion, our study suggests that dietary ω-3 and ω-6 fatty acids intake might be inversely associated with low cognitive performance for participants aged 60 years or older in the U.S. The associations we investigated in this study are biologically plausible, and these findings are required to be confirmed by further large-scale prospective studies.

## Data Availability

The datasets supporting the conclusions of this article are available in publicly repository as described below. The authors do not own the data. National Health and Nutrition Examination Survey data are available from the National Center for Health Statistics (http://www.cdc.gov/nchs/nhanes/nhanes_questionnaires.htm).

## Abbreviations

PUFAs: Polyunsaturated fatty acids
NHANES: The National Health and Nutrition Examination Surveys
CERAD: The Consortium to Establish a Registry for Alzheimer’s disease
DSST: The Digit Symbol Substitution Test
GPAQ: The Global Physical Activity Questionnaire
PIR: Poverty income ratio
HHS: The Department of Health and Human Services
MEC: Mobile examination center
PFU: Phone follow-up
BMI: Body mass index
OR: Odds ratio
CI: Confidence interval
